# Effectiveness of combined intermittent preventive treatment for children and timely home treatment for malaria control

**DOI:** 10.1186/1475-2875-8-292

**Published:** 2009-12-11

**Authors:** Collins K Ahorlu, Kwadwo A Koram, Atsu K Seakey, Mitchell G Weiss

**Affiliations:** 1 Noguchi Memorial Institute for Medical Research, University of Ghana, Box LG581, Legon, Ghana; 2 Keta District Health Management Team, Box KW198, Keta, Ghana; 3 Swiss Tropical Institute, Socinstrasse 57, CH-4002, Basel, Switzerland

## Abstract

**Background:**

Whiles awaiting for the arrival of an effective and affordable malaria vaccine, there is a need to make use of the available control tools to reduce malaria risk, especially in children under five years and pregnant women. Intermittent preventive treatment (IPT) has recently been accepted as an important component of the malaria control strategy. This study explored the potential of a strategy of intermittent preventive treatment for children (IPTC) and timely treatment of malaria-related febrile illness in the home in reducing the parasite prevalence and malaria morbidity in young children in a coastal village in Ghana.

**Methods:**

The study combined home-based delivery of IPTC among six to 60 months old and home treatment of suspected febrile malaria illness within 24 hours. All children between six and 60 months of age received intermittent preventive treatment using amodiaquine and artesunate, delivered by community assistants every four months (three times in 12 months). Malaria parasite prevalence surveys were conducted before the first and after the third dose of IPTC.

**Results:**

Parasite prevalence was reduced from 25% to 3% (p < 0.00, Mann-Whitney) one year after the inception of the two interventions. At baseline, 13.8% of the children were febrile (axillary temperature greater than or equal to 37.5 degree Celsius) compared to 2.2% at evaluation (post IPTC3 combined with timely home management of fever) (p < 0.00, Mann-Whitney).

**Conclusion:**

The evaluation result indicates that IPTC given three times in a year combined with timely treatment of febrile malaria illness, impacts significantly on the parasite prevalence. The marked reduction in the parasite prevalence with this strategy points to the potential for reducing malaria-related childhood morbidity and mortality, and this should be explored by control programme managers.

## Background

Malaria is estimated to cause between 300 and 500 million clinical cases with about 700,000 to 1.6 million deaths every year. About 94% of these deaths are believed to occur in sub-Saharan Africa, which is also the most resource-limited continent on the globe [1,2]. Malaria remains a major cause of morbidity and mortality in Ghana, accounting for over 40% of outpatient clinic visits and about 20% of deaths in children under five years of age [3]. The use of artemisinin-based combination therapy (ACT) for the treatment of uncomplicated falciparum malaria has been shown to provide effective treatment against falciparum malaria and slow down the spread of drug resistance [1]. In line with this recommendation and the increasing treatment failure rate of chloroquine [4], the Ghana National Malaria Control Programme, (GNMCP) has adopted the use of artesunate and amodiaquine for the treatment of uncomplicated malaria in the country since January, 2005. This became necessary because by 2003 parasitological responses to chloroquine were less than 50% in some areas of the country. Similar studies on the efficacy of sulphadoxine/pyrimethamine (SP) have shown 0-36% of RII resistance and 0-9% of RIII resistance [5,6].

Malaria control in Ghana relies on early diagnosis and prompt treatment of suspected cases (fevers) and the home is where early recognition and, in most cases, prompt treatment is initiated [7,8]. However, the current combination therapy is not widely available for home management. Reluctance to make combination therapy available for home management results from concerns that making these drugs widely available may lead to abuse and, therefore, increased drug pressure on the parasites which could lead to the emergence of *Plasmodium falciparum *resistance to these anti-malarial drugs, as with chloroquine and sulphadoxine/pyrimethamine.

Prompt and efficacious treatment of malaria cases is an effective strategy for malaria control. It is especially important for vulnerable groups including children under five years of age and pregnant women. IPT has also recently been accepted as an important component of the malaria control strategy following the demonstration, in perennial malaria transmission areas, that IPT given with childhood vaccinations reduced incidence of first episode of malaria. It also reduced severe anaemia by more than 50% during the first year of life [9-11]. IPTC studies were carried out in areas with seasonal malaria transmission and were found to be effective [9,12]. The use of bed net is one of the mainstays of malaria control in Africa and to promote the use of treated bed net, the Ghana Health Service has made them available at community levels. These bed nets were highly subsidized for children under five years and pregnant women.

### Rationale

Although the Abuja target of treating 60% of under-five suspected malaria cases within 24 hours of symptom onset by 2005 was reduced by the Ghana Health Services to 55% in 2007, this goal has not been achieved, and the question remains as to how this will be achieved. Improved access to IPTC especially in communities with no health facilities is one way to reduce the prevalence of infection and associated morbidity. IPTC studies were carried out in areas with seasonal malaria transmission and were found to be effective [9,12], but the question is will it be effective in areas with perennial malaria transmission, especially when combined with timely home management? This study was designed to test the potential of a strategy of IPTC delivered at home and timely treatment of febrile illness to reduce parasite prevalence and malaria morbidity in children under five years of age, in rural coastal Ghana.

### Objectives

The goal of this study was to demonstrate the feasibility of controlling febrile malaria illness at the community level through combined interventions of intermittent preventive treatment and timely febrile malaria illness management in children between six and 60 months of age in southern Ghana. Specifically, the study was designed to: deliver intermittent preventive treatment to children (IPTC) at home and to provide timely treatment for febrile malaria illness in children within 24 hours of symptom onset.

## Methods

### Study sites

The study was conducted at Shime sub-district of the Keta district in the Volta region of Ghana where malaria accounts for over 40% outpatient clinic attendance in the district (District Annual Report, 2006). The malaria situation in the district is perennial with higher transmission occurring in March (the beginning of the raining season) to November (the end of the raining season). The Shime sub-district is one of the four administrative sub-districts recognized by the Ghana Health Service in the Keta District. It is the most deprived area in the Keta district. Electricity supply is limited; it has no secondary school and a very poor road network. During the rainy season most parts of the area are cut off from the rest of the district. The land is marshy and covered with streams, lagoons and creeks. The entire sub-district has a population of 19,972. The study took place in an area with a population of 8,000. About 99% are Anlo-ewe-speaking people. Their main occupation is farming and fishing.

The sub-district is served by two health centers and one of them is located within the study area. Almost all the communities in the area have pipe-borne water, but because of the 'cash and carry system' where residents pay for every bucket of water fetched at the point of fetching, the people continue to use unsafe water from the many water bodies around to complement the safe one. Four out of the 36 communities in the sub-district participated in the study, mainly because of geographical accessibility.

### Study participants

Community entry activities, involving meetings and durbars of chiefs and people in the study communities, were done at the onset of the project. After gaining the support and willingness of the chiefs and people to participate in the study, the community registers of eligible children aged ≤60 months were updated. After obtaining consent from caretakers/parents, children aged six to 60 months were enrolled in the study. No caretaker refused to participate. All children (six to 60 months old), including those on antiretroviral treatment, were included in the study.

### Questionnaire survey

All caretakers of children selected to participate in the baseline parasite survey were selected as respondents in a semi-structured questionnaire interview. The questionnaire was designed to solicit information on the demography of caretakers, all cause mortality, morbidity, bed net usage among others. This short interview guide was translated into the local language and back translated into English to ensure that they convey the meanings they were intended to. The questionnaire was pre-tested in a field situation in a community with similar characteristics as the study community. The interview was conducted in the local Ewe language by the first author.

### Intermittent preventive treatment for children

IPTC was delivered every four months beginning in July 2007 for one year (July, November 2007 and March 2008) and the strategy was evaluated in July, four months after the last round of IPTC and just before the fifth round of IPTC. Children received 10 mg/kg body weight of amodiaquine (AQ) daily and 4 mg/kg of artesunate (AS) daily (given as a single dose) over three days. All treatments were given under direct observation and children were observed for five minutes after drug administration to ensure that they retained the medication. Children who vomited within the five-minute observation period received a repeated full dose of the medication. Children were followed up on days 1, 2, 3 and 7 after treatment (the day of first dose (treatment) was counted as Day 0) to document adverse events reported by caretakers using the adverse report form.

### Timely treatment of suspected febrile malaria in children

Any child with febrile illness suspected to be malaria by the caretaker was reported to the fieldworkers who then evaluated and treated those who met the protocol criteria for suspected febrile malaria illness. Those who did not meet the criteria were referred to the health post located within the sub-district for further evaluation and treatment. In short, any child with axillary temperature of ≥37.5 degree Celsius, whose caretaker suspected febrile malaria illness, was treated with the full three-day course of artesunate and amodiaquine.

### Laboratory methods

A diagnosis of malaria infection was made by light microscopy of thick and thin blood smears. Finger print peripheral blood was taken by trained technologists from the Noguchi Memorial Institute for Medical Research for the preparation of the thick and thin blood smears. All specimens were taken from the field. The thick and thin blood smears were stained with 3% Giemsa for 30 minutes. Parasite density was determined by counting the number of asexual parasites per 200 white blood cells, and calculated per μL assuming a white blood cell count of 8,000 cells per μL. Sexual parasite count was done per 1,000 white blood cells. A smear was declared negative when the examination of 100 thick-film fields did not reveal the presence of asexual parasites. Quality control was done by allowing a second microscopist to read a random 10% sample of both negative and positive slides to confirm the absence or presence of parasites. Discrepant results were read by a 3^rd ^microscopist and the majority result was taken as the final result. Haemoglobin concentration was determined from finger pricks using a portable automated Hemocue^® ^photometer (Leo Diagnostics, Sweden) at the field site.

### Sample size

Every qualified person living in the study community was enrolled with the consent of his or her caretaker, mostly parents. However, for the prevalence survey, a sample size of 174 was needed to determine malaria parasite prevalence estimated at 20% in the study population. Also, to be able to determine at least10% reduction in malaria parasite prevalence in the study population at evaluation, we needed to enrol about 360 children into the IPTC intervention programme [13]. At evaluation however, after examining 174 children, randomly selected, and detecting very low parasite prevalence, all available participants at the time of the evaluation were examined. This led to the examination of over 80% of the children enrolled into the study.

### Data analysis

Data were analysed to compare pre-intervention findings with post-intervention findings. All quantitative analyses were done using EpiInfo version 3.4.1. Proportions of pre- and post-intervention clinical findings and parasite levels were compared using Mann-Whitney or chi-squared tests. Statistical significance was set at p ≥ 0.05.

## Results

### Caretakers' interviews

The mean (± SD) age of respondents was 28.2 (± 7.5) years. Most, (71.0%) of respondents were married. The mean (± SD) years of formal education was 3.9 (± 3.6) ranging from 0 to 12 years. However, 37.1% of respondents had no formal education.

Respondents (98.0% for baseline and 100% at evaluation) reported that they and their families usually sleep under bed net. Comparatively, the number of insecticide-treated net usage has increased significantly from 38.5% to 60.0% in the study community within one year of project implementation. This was as a result of continual information provided by community assistants to caretakers that treated bed net was an effective way to reduce febrile malaria illness, and also participants were encouraged to take advantage of a highly subsidized treated bed net provision programme available at the health facility for children under five years of age and pregnant women. All respondents (baseline and evaluation) said it was important for children under five years to sleep under bed nets.

The mortality data generated was too small to make any meaningful analysis, but it is important to report that, at baseline, five (3.0%) caretakers reported that they had lost a child within the 12 months preceding the interview. The conditions that killed these children were reported as *Asra *- febrile malaria (2), convulsion (1), *swelled up *(1) and *could not tell *(1). Also, 35 (20.1%) respondents said they knew a close relative who had lost a child less than five years of age within the past 12 months at baseline. No caretaker, however, had lost a child or had a relative that had lost a child in the twelve months of project implementation.

### Parasite prevalence and haemoglobin surveys

During the prevalence surveys (baseline and evaluation) a number of febrile illnesses were reported for the children (within the seven days prior to data collection) (Table 1). On the day of examination, 42% (baseline) and 7.6% (evaluation) of caretakers reported that the children had fever within the past seven days, an approximate reduction of 80% in the point prevalence of fever cases in the study community (p < 0.00, Mann-Whitney test).

**Table 1:** The distribution of febrile illnesses reported for children within the past seven days prior to the baseline and evaluation prevalence surveys. **

Variables	Baseline (N = 174)	Evaluation (N = 357)
Chills and Rigors	18 (10.3)	13 (3.6)*
Convulsions	4 (2.3)	0*
Diarrhoea	32 (18.4)	15 (4.2)*
Fever/Malaria/Asra	73 (42.0)	27 (7.6)*
Headache	58 (33.3)	18 (5.0)*
Others (Cough, Rashes, Stomach ache etc.)	20 (11.5)	8 (2.2)*
Vomiting	20 (11.5)	11 (3.1)*

** Sorted in column 1 in alphabetical order.

* P. value < 0.00

At baseline, five (2.9%) caretakers reported that the children were treated for febrile malaria within the seven days prior to data collection. These five were reportedly treated with anti-malarials, such as chloroquine, *malaherb *(local herbal-based anti-malarial preparation) and Kinaquine^® ^(a locally-manufactured brand of chloroquine). None of these five children were taken to the clinic/hospital. On the other hand, during the evaluation, only two caretakers reported that their children had been treated within the seven days prior to data collection and they were treated by the community assistants working with the project using amodiaquine and artesunate. However, community assistants' have referred a total of 29 children whose conditions they suspected not to be febrile malaria-related-mostly children having running nose/catarrh, stomach problems, ear infections, eye problems, watery diarrhoea and fast breathing among others, to the health post (clinic) for further investigations and treatment within the 12 months of study implementation.

### Parasite prevalence

Out of 174 children tested for malaria parasites at baseline, 44 (25%) of them were positive for malaria parasite compared with only 10 (3%) out of 357, a significant difference of over 80% (p < 0.00, Mann-Whitney test). All the positive infections were *P. falciparum*, the dominant species in Ghana. Detailed clinical and parasitological findings are presented in Table 2. One child (evaluation) with a very high parasite density was found to have been taken out of the study community after receiving the first IPTC and brought back to the community a few days before the evaluation survey.

**Table 2:** Baseline and Evaluation Characteristics, Clinical and Parasitological findings

Variables	Baseline (N = 174)	Evaluation (N = 357)
Mean age in months (± std.)	30.5 (16.5) months	37.7 (15.8) months
Sex (% of female)	52.3	47.0
Weight: Mean (± std.)	12.3 (3.5)	13.8 (3.5)
Haemoglobin < 10 g/dl	48 (27.6)	60 (16.8)
Number (%) febrile (axillary temp ≥ 37.5°C)	24 (13.8)	8 (2.2)
Parasite prevalence number (%)	44 (25.3%)	10 (3.0)
Geometric mean parasite density	235.30	306.33)
Minimum (maximum) pasitaemia level	30 (900)	30 (29820)

± std. = standard deviation

### Anaemia

In this study, anaemia was defined as haemoglobin < 10 g/dl. There was a significant drop of anaemia in the study population one year into the intervention (p < 0.004, Mann-Whitney test), at baseline, 27.6% of the children were anaemic compared to 16.8% recorded during evaluation (table 2).

There was no relationship between gender and anaemia in the study population. There was a significant relationship between reported fever in the seven days prior to baseline survey and anaemia (X^2 ^= 5.5309, p = 0.01), but this was not the case at evaluation. Also, at baseline there was a significant relationship between parasitaemia and anaemia (X^2 ^= 5.2028, p = 0.02), but this was not the case at evaluation. There was a significant relationship between febrile status and anaemia at evaluation (P = 0.01 (Fisher exact test)), but this was not the case at baseline.

There was a significant relationship between febrile status and parasitaemia at both baseline and evaluation (X^2 ^= 49. 3643, p = < 0.00 - baseline; p = < 0.00 (Fisher exact test)) - evaluation). There was no gender difference in parasitaemia in the study population.

### Intermittent preventive treatment for children (IPTC)

IPTC was delivered to children aged six to 60 months in the study community three times (July and November 2007 and March 2008). There have been marginal increases in the number of children at each subsequent treatment round (IPTC1 = 413, IPTC2 = 420 and IPTC3 = 433) (Table 3). Only 22 and 18 children have received IPTC twice and once respectively at the time of evaluation. This was due largely to the movement of these children out of the study community. However, the number of those who received IPTC once or twice was too small that we could not perform any meaningful comparative analysis between them and those who received IPTC for three times.

**Table 3:** The distribution of febrile illnesses reported for children within the past seven days prior to each drug administration. **

Febrile illness reported in the past seven days prior to drug administration	IPTC1 (N = 413)	IPTC2 (N = 420)	IPTC3 (N = 433)
	Number (%)	Number (%)	Number (%)
Fever/Malaria/*Asra*	64 (15.50)	43 (10.20)	11 (2.5)
Headache	49 (11.90)	39 (9.30)	5 (1.2)
Vomiting	25 (6.10)	11 (2.60)	1 (0.2)
Chills and Rigors	17 (4.10)	10 (2.40)	7 (1.6)
Others (Cough, Rashes, Stomach ache etc.	7 (1.70)	5 (1.20)	9 (2.1)
Convulsions	5 (1.20)	0	0
Diarrhoea	5 (1.20)	1 (0.20)	3 (0.7)

** Sorted in column 2 in descending order.

During each IPTC round, a number of febrile illnesses were reported by caretakers within the past seven days prior to treatment (Table 3). On the day of treatment 15.50% (IPTC1), 10.20% (IPTC2) and 2.50% (IPTC3) respectively of caretakers reported that the children have had fever within the past seven days. This shows a steady drop in fever cases in the study community at each subsequent treatment.

There was virtually no serious adverse event reported after drug administrations. However, 5 (1.21%) IPTC1, 4 (0.95%) IPTC2 and 7 (1.65%) IPTC3, caretakers reported that their children were weak for about two to four hours after taking the drugs. None of these cases warranted any medical intervention.

Only five (1.2%) febrile malaria-related illnesses were treated in the study communities between IPTC1 and IPTC2, while 39 (9.3) were treated between IPTC2 and IPTC3 and 17 (3.9%) were treated between IPTC3 and evaluation.

### Informal information, education and communication

Throughout the 12-month intervention period, community assistants continued to informally advice community members on the importance of IPTC, the need for timely treatment of suspected febrile malaria illness in children within 24 hours of the onset of symptoms and the need to replace the predominantly untreated bed nets with treated ones. Community members were encouraged to take advantage of the highly subsidized treated bed nets available at the health centre for children under five years and pregnant women. Pregnant women were also encouraged and in some cases assisted to attend antenatal clinics.

### Feasibility of combining IPTC and timely home management for malaria control

The study clearly demonstrates that it is possible to train community assistants to deliver IPTC and timely home treatment to children aged six to 60 months. Visits were made to the community twice in a month to collect timely home treatment forms. However, during each IPTC rounds, the first author spent three days in the community to supervise the research assistants.

## Discussion

This study looked at the effectiveness of intermittent preventive treatment for children (IPTC) combined with timely treatment at home for malaria control, targeting children aged six to 60 months old in an all year round malaria endemic area in Ghana. The main finding of the study was a reduction of about 88.0% in the prevalence of malaria parasite infections in the target population (from 25.0% at baseline to 3.0% at evaluation) within one year of the project implementation. It may be argued that the increase in the use of insecticide treated net (from 38.5% to 60.0%) could be implicated in the reduction of the malaria parasite prevalence in the study population. However, it is unlikely that all the reduction of over 80.0% was due to the 55.8% increase in ITN use alone. Several studies in IPT intervention measured clinical incidence rather than prevalence and found between 20% and 86.0% reduction with strong variations depending on transmission duration and intensity, target population and intervals between treatments [9-12,14,15]. In this study, prevalence was measured because it is easier to measure as the interventions were delivered by community assistants. The second reason for measuring prevalence was the combination of IPTC with home treatment, which requires that suspected cases were treated once they meet the inclusion criteria and for ethical reasons, the community assistants were not trained to take blood for examination before treating the children. However, this may be seen as a weakness of the study.

Anaemia in the children, defined as haemoglobin <10 g/dl, improved from 27.6% to 16.8%, 12 months after the intervention was implemented. This compares well with a recent randomized trial in Tanzania, which showed that IPT given to infants at the time of childhood immunization reduced the incidence of the first episode of malaria and anaemia by more than 50.0% during the first year of life [10,11].

There was a noticeable reduction in malaria-related morbidity in the study population as expressed by fever and other malaria-related signs and symptoms reported either in the seven days before prevalence surveys or IPTC administration. The reduction could also be seen from reported treatment sought for the children within the seven days prior to prevalence surveys or IPTC administration. Timely treatment of febrile malaria cases in the community did not follow any pattern as can be seen from the result. However, it could be argued that because the community assistants were on hand to deliver effective treatment in a timely fashion, this could have contributed to the marked reduction of parasitaemia seen at the evaluation survey. The community assistants also contributed to heightened health information in the study community through informal education on the use of treated bed net and timely treatment. This should encourage malaria control programmes to have confidence in community assistants to deliver timely treatment and IPTC to children at the community level, once they are well trained by the programme coupled with reference manual for easy and quick referencing when in doubt.

Findings reported here present a challenge to the existing practice, especially in most sub-Saharan African countries, where malaria diagnosis is based on presumption without confirmation. As community intervention or treatment increases, this may lead to fewer malaria infections and this may lead to over diagnosis and treatment with expensive drugs for people who do not need them. When this happened, control programmes would have to invest in rapid diagnostic test kits where microscopy is not possible. As reported by Zikusooka *et al *[16], this may lead to cost savings because anti-malarial drugs are expensive. Goodman *et al *[17] also make this point. Furthermore, rational use of anti-malarials will reduce the potential for adverse reactions.

The sharp increases in the number of febrile malaria cases treated at home between IPTC2 and IPTC3 might have been largely due to increased confidence of caretakers in the ability of community assistants to treat their children with suspected febrile malaria effectively and the recognition of caretakers of the importance of timely treatment. The decrease in the numbers between IPTC3 and evaluation could be attributed to the reduction of malaria prevalence in the study community as a result of the interventions implemented.

The use of IPTi which is similar to IPTC in principle was found to reduce malaria incidence in infants [10]. Although this study cannot determine the contribution of IPTC and timely treatment at home to the protection offered to the children because the two interventions were delivered concurrently, the two together in this study offered a major protection against malaria in children, reducing prevalence from 25% to 3%.

The results indicate that it is possible to deliver IPTC and timely home treatment to children between six and 60 months old. Since there were no timely treatment form to collect at some of the biweekly visits to the community, it should be possible to reduce the visit to once in a month to reduce supervision cost.

This indicates that, it is possible to reduce malaria prevalence and this may reduce malaria-related childhood morbidity and mortality and this should be explored by control programme managers as one of the effective options available for the fight against malaria, especially in sub-Saharan Africa.

## Competing interests

The authors declare that they have no competing interests.

## Authors' contributions

CKA was involved in the conception, design and implementation of the study as well as data management and writing of this paper. KAK was involved in the conception, design and writing of this paper. AKS was involved in the design, implementation and writing of this paper. MGW was involved in the conception, design and writing of this paper. All the authors have read and approved the final version of the manuscript.

## Ethical review

The study was approved by the Institutional Review Board of the Noguchi Memorial Institute for Medical Research, University of Ghana and the Ethical Review Committee of WHO/TDR.
